# Polymorphism of the *MTNR1A* Melatonin Receptor Gene in Goat Breeds of Northern Italy

**DOI:** 10.3390/ani13243855

**Published:** 2023-12-15

**Authors:** Stella Agradi, Sebastiano Luridiana, Maria Consuelo Mura, Giovanni Cosso, Daniele Vigo, Laura Menchetti, Gabriele Brecchia, Vincenzo Carcangiu, Giulio Curone

**Affiliations:** 1 Department of Veterinary Medicine and Animal Sciences, University of Milan, Via dell’Università 6, 26900 Lodi, Italy; stella.agradi@unimi.it (S.A.); daniele.vigo@unimi.it (D.V.); giulio.curone@unimi.it (G.C.); 2 Department of Veterinary Medicine, University of Sassari, Via Vienna 2, 07100 Sassari, Italy; sluridiana@uniss.it (S.L.); mcmura@uniss.it (M.C.M.); gicosso@uniss.it (G.C.); endvet@uniss.it (V.C.); 3 School of Biosciences and Veterinary Medicine, University of Camerino, Via Circonvallazione 93/95, 62024 Matelica, Italy

**Keywords:** local breed, melatonin receptor gene, seasonal reproduction, small ruminants

## Abstract

**Simple Summary:**

To date, there is a lack of available data regarding Italian goat breeds other than the Sarda goat in relation to the polymorphism of the *MTNR1A* melatonin receptor gene, which is responsible for variations in the reproductive performance of small ruminants. The objective of this study was to detect any PCR-RFLP polymorphic sites of *MTNR1A* in Northern Italian goat breeds, notably known for a pronounced reproductive seasonality. Unexpectedly, no polymorphism was detected in any of the investigated goat breeds. It is hypothesized that the absence of polymorphism may be linked to the macro group of goat breeds that evolved during human migrations. Specifically, breeds of the Alpine–European strain would appear to show no polymorphism, as confirmed by our study, whereas breeds belonging to the Mediterranean–African or Asian–Middle Eastern strains do. Future investigations will be needed to confirm this hypothesis and clarify the role of *MTNR1A* in regulating the reproductive activity in goats.

**Abstract:**

Melatonin receptor MT1, encoded by the *MTNR1A* gene, is the main one involved in the seasonal regulation of reproductive activity. A correlation between this gene polymorphism and reproductive performance has been demonstrated in sheep. To date, no data about *MTNR1A* gene polymorphism are available regarding Italian goat breeds other than the Sarda goat. This study aimed to detect any PCR-RFLP polymorphic sites of *MTNR1A* using *MnlI* and *RsaI* enzymes in Northern Italian goat breeds, which are characterized by a pronounced reproductive seasonality. One-hundred-eight adult female goats belonging to four different breeds were included in the study (i.e., Frisa Valtellinese, *n* = 29; Orobica, *n* = 23; Lariana, *n* = 29; Camosciata delle Alpi, *n* = 27). Blood was sampled from each goat. Genomic DNA was extracted from each sample and the main part of exon II of *MTNR1A* gene was amplified by PCR and digested with *MnlI* and *RsaI* enzymes. Unexpectedly, none of the fragments were found to be polymorphic. The absence of polymorphism may be linked to the macro group of goat breeds that evolved during human migrations. Breeds of the Alpine–European strain would appear to show no polymorphism, as confirmed by our study, whereas breeds belonging to the Mediterranean–African or Asian–Middle Eastern strains do.

## 1. Introduction

Melatonin is a hormone that was first isolated and characterized from the bovine pineal gland by dermatologist Aaron Lerner in 1958. Melatonin derives its name from its ability to induce the lightening of frog skin through the contraction of melanophores and the fact that it is synthesized from a serotonin molecule [1]. Melatonin has been subsequently identified in all major taxonomic groups, including both invertebrate and vertebrate species. Its synthesis occurs in the mitochondria (or chloroplasts and prokaryotes outside the Animalia kingdom), indicating that it is one of the phylogenetically oldest signaling mechanisms according to the evolutionary theory of endosymbiosis [2]. In vertebrates, melatonin is primarily secreted by the pineal gland, and secondarily by the retina, gastroenteric system, skin, platelets, lymphocytes, bone marrow, and likely other structures as well [3]. Melatonin possesses amphiphilic properties, granting it the ability to easily diffuse across cell membranes, in particular, the blood–brain barrier [2]. In higher organisms, it plays a key role in regulating circadian rhythms and reproductive seasonality. The latter function is based on the conversion of information from the photic environment, indicating the duration of day and night, into a chemical signal, namely melatonin and its metabolites [2,4]. The synthesis pathway of this molecule is regulated at multiple levels by the light signals received by the retina [5]. Specifically, the secretion of melatonin by pinealocytes reaches its peak during the nighttime, in the dark hours [3]. For this reason, melatonin secretion varies throughout the year, allowing for an adaptive response to environmental changes [6].

Melatonin plays a crucial role in the regulation of reproductive activity in small ruminants. Indeed, these animals are classified as short-day breeders, as the resumption of their reproductive activity coincides with the shortening of the photoperiod [7] and is determined by the melatonin-mediated activation of the hypothalamic–pituitary–gonadal axis, which is suppressed during the anoestrus season [7]. In fact, the increase in hours of darkness corresponds to an increase in melatonin secretion, which, through pathways not yet fully elucidated, results in an increase in tonic GnRH secretion at the hypothalamic level [8,9]. The increased pulsatile release of GnRH is subsequently responsible for the release of luteinizing hormone (LH) and follicle-stimulating hormone (FSH) by the pituitary gland. These hormones then reach the gonads and induce the resumption of the gonadal activity [8]. Therefore, in small ruminants, during the reproductive season, longer peaks of melatonin will occur, as the number of hours of darkness that trigger melatonin synthesis increases during this season [8]. As above mentioned, the synthesis and secretion of melatonin are regulated at several levels, and, in particular, the activity of the enzymes involved in its synthetic pathway is closely linked to the light impulses that reach the retina [10]. To date, melatonin has been demonstrated to have various physiological actions in addition to the regulation of reproductive activity. However, these actions are still under investigation and depend on the types of receptors with which melatonin interacts once released into the bloodstream, as well as their localization in the organism. In mammals, there are two melatonin receptors, MT1 and MT2, both of which are G protein-coupled receptors. In particular, MT1 has been identified in different tissues, including the *pars tuberalis* of the pituitary gland and the hypothalamus [4]. Studies indicate that this receptor is the main one involved in the seasonal regulation of reproductive activity [11,12]. Moreover, melatonin also acts through mechanisms not mediated by receptors; for example, it acts as an antioxidant molecule [3]. On the other hand, the MT receptor-mediated-effects of melatonin on the hypothalamic–pituitary–gonadal axis can be manipulated both pharmacologically and through light regulation mechanisms in order to anticipate the reproductive season and induce cyclicity in small ruminants [13], as well as to advance the attainment of puberty [14]. Finally, research in recent years has investigated other non-reproductive effects of melatonin. A surprisingly wide range of other effects on the organism have been demonstrated, and pharmacological treatment with synthetic melatonin has often proved useful in regulating them. These effects include antioxidant properties [15], seasonal molting [16,17], temperature and cardiovascular regulation [18], immunomodulation [19], microbiota regulation [19], and regulation of skeletal muscle growth and development, which all have significant consequences on the productive and reproductive performance of small ruminants [20].

The MT1 receptor is encoded by the *MTNR1A* gene [21]. This gene has been mapped in several species, including humans, mice [22], swine, and bovine [23]. Several polymorphic sites have been recognized in this gene, considering different species such as sheep, goats, and buffalo [23,24]. The ovine *MTNR1A* gene polymorphic RFLP sites were first identified and described by Messer et al. in 1997 [23]. In this study, two polymorphic sites were shown and detected using *MnlI* and *RsaI* enzyme digestion [23]. Following this discovery, different studies in sheep have been conducted to investigate the existence of any relationship between *MTNR1A* gene polymorphism and variation in reproductive performance [25,26,27,28,29,30,31,32,33,34,35]. Specifically, different investigations have shown the presence of a polymorphic cleavage site in the g.17355452 (G > A) position, evidenced by the *MnlI* enzyme, and in the g.17355458 (C > T) position, evidenced by the *RsaI* enzyme [36,37]. The presence of the cleavage site in g.17355452 was identified with the allele known as M and the absence known as m, while the presence of the cleavage site in g.17355458 was identified with the allele known as R and the absence known as r [38]. In sheep, both M/M and R/R genotypes have been associated with non-seasonal reproductive activity [25,37,39,40,41]. In particular, different studies conducted on Sarda and Rasa Aragonesa sheep have shown the association between the *MTNR1A* gene polymorphism and the period of reproductive resumption, the length of the reproductive season, the number of days from ram placement with ewes to lambing, the fertility, and the reproductive response following the synchronization and artificial insemination in the spring [42,43,44,45]. Thus, in summary, the investigations on *MTNR1A* gene polymorphism have shown an association between this gene and the modulation of seasonal effects on the reproductive activity of sheep.

Nevertheless, research on this matter in goats remains limited. The first investigation on the goat *MTNR1A* polymorphism was conducted in 2002 and evidenced the existence of seven *MnlI* sites, yet none showed polymorphism [46]. Since this study, others have been conducted on different goat breeds [38,47,48,49,50,51]. In general, no polymorphisms have been identified by the *MnlI* restriction enzyme, with all the goat breeds investigated showing an M/M genotype. For *RsaI* enzyme, only two genotypes have been identified to date: R/R and R/r. Specifically, the R/R genotype is the most common, whereas the R/r genotype was found in only a limited number of studies and within a small proportion of the investigated goat breed populations [38,46,47,48,49,50,51]. Regarding the relationship between gene polymorphism and reproductive activity, studies remain scarce and contradictory. However, a limited number of investigations, including those conducted on the Sarda goat, suggest an association between the R/R genotype and a year-round estrus reproductive pattern, and, on the other hand, a link between the R/r genotype and a strong influence of the photoperiod on reproductive activity in goats [38,47]. However, to date, no data are available about Italian goat breeds other than the Sarda goat, which is a local breed typical of the Sardinia region.

In the Italian Alps, the latitude and, consequently, the photoperiod, are different from those of the Sardinia region. Frisa Valtellinese, Orobica, and Lariana are three native goat breeds from Northern Italy, which have been bred for centuries in the Alps and are characterized by pronounced reproductive seasonality with a birth season running from late December to early March. The Camosciata goat, on the other hand, is a cosmopolitan breed native to the Swiss Alps, which exhibits the same seasonality as the above-mentioned native breeds at similar latitudes. These breeds belong to the same breed cluster. Indeed, the spatial and temporal distancing from the domestication center of the goat at the beginning of the Holocene epoch caused genetic divergence among the first goat populations, leading to the differentiation of specific breeds within three different clusters: the Asian cluster, the European cluster, and the African cluster [52]. The local breeds of Italy belong to the European cluster, whereas the greater part of the breeds investigated for *MTNR1A* gene polymorphism belong to the Asian one. The hypothesis of this study is that the Alpine-arch native goat breeds, which have never been investigated, are genetically different from the Asian goat breeds, and may show significant differences in the allele frequencies of the *MTNR1A* gene compared to what has been reported in the scientific literature for year-round estrous breeds [47]. This study aimed to detect any PCR-RFLP polymorphic sites of *MTNR1A* using *MnlI* and *RsaI* enzymes in Northern Italian goat breeds.

## 2. Materials and Methods

### 2.1. Animals Involved and Sample Collection

In this study, a total of 108 adult female goats belonging to four different breeds were included (i.e., Frisa Valtellinese, *n* = 29; Orobica, *n* = 23; Lariana, *n* = 29; Camosciata delle Alpi, *n* = 27). Frisa Valtellinese or Frontalasca goats, Orobica or Val Gerola goats, and Lariana or Di Livo goats are local breeds that have been bred for centuries in different valleys of Northern Italy in the Lombardy Alps. Studies on these autochthonous breeds are very limited, and they are facing extinction because of the progressive abandonment of both their farming and the use of mountainous lands where they are native. Camosciata delle Alpi or the Alpine goat is a transboundary breed native to Switzerland, mainly bred to produce milk. All four breeds involved in this study are characterized by a kidding season that typically extends from late December to early March. In Northern Italian human societies, this seasonality has historically been associated with the traditional consumption of goat kid meat during the Easter holidays.

The animals involved in this study were randomly selected from four different farms located in Lodi Province for Camosciata delle Alpi goats, in Sondrio Province for Frisa Valtellinese and Orobica goats, and in Como Province for Lariana goats, which had about 100 heads per stable. Thus, the farms involved were all located in the Lombardy region between the 45 and 46 north latitude parallels. The management of the Frisa Valtellinese, Orobica, and Lariana goat farms was the same, based on a traditional semi-extensive farming system for the production of cheese and kid meat. Camosciata delle Alpi goat was bred for the production of both cheese and milk following a semi-intensive farming system. However, all the goats were kept under a natural photoperiod since birth. Males were introduced in the flocks from mid-July until early November. Before inclusion in the investigation, all goats were clinically examined, and only healthy animals were enrolled. Only pluriparous goats were included in this study, and the mean age (and standard deviation) was 5.7 ± 2.7, 5.8 ± 1.8, 5.3 ± 1.8, and 3.1 ± 1.1 years for Frisa Valtellinese, Orobica, Lariana, and Camosciata delle Alpi goats, respectively. The number of kids born to each goat breed included in this study for the kidding season of the year under investigation is reported in Table 1.

The kidding season occurred between the following timeframes: from 16 December to 4 January for Frisa Valtellinese goats, from 14 February to 23 March for Orobica goats, from 21 December to 8 January for Lariana goats, and from 11 January to 17 February for Camosciata delle Alpi goats.

All animals included in this study were examined beforehand by an experienced veterinarian, and only clinically healthy animals were included in this study. Ten mL of blood was sampled from each goat by jugular venipuncture using 18G disposable needles into sterile vacuum tubes containing K3EDTA as an anticoagulant (BD Vacutainer Systems, Franklin Lakes, NJ, USA). Blood samples were then stored at −20 °C until analysis. The collection of blood samples was performed with a strict commitment to animal welfare in accordance with the current Italian legislation. This study was conducted with the approval of the Institutional Animal Care and Use Committee of the Università degli Studi di Milano (Permission OPBA_04_2021).

### 2.2. Genomic DNA Preparation and Primers Sequences

Genomic DNA was extracted from whole blood using a commercial kit (Purgene, Gentra, Minneapolis, MN, USA) and stored at −20 °C until further analysis.

For the polymerase chain reaction (PCR), 100 ng of genomic DNA was used with primers by Messer et al. [23], synthesized by Sigma Genosys Ltd. (Pampisford, Cambs, UK). The primers were of the sequence of Reppert et al. [53], which are part of exon II of the MTNR1A ovine gene (GenBank U14109), from position 285–304 [38,46,47] (sense primer: 5′-TGT GTT TGT GGT GAG CCT GG-3′) and 1108–1089 (antisense primer: 5′-ATG GAG AGG GTT TGC GTT TA-3′). Fifty μL of total volume was used to start PCR. Specifically, the starting volume contained 5 μL of 10× PCR buffer (which was a solution composed of 50 mM L/L KCl, 10 mM Tris-HCl (pH 8.0), 0.1% Triton X-100), 3 μL of 1.5 mM MgCl_2_, 8 μL of 0.2 mM solution containing each dNTP, 1 μL of 10 pM/L solution containing each primer, 100–150 ng of extracted genomic DNA, and 5 U of Taq DNA polymerase (HotMaster Taq DNA Polymerase, Eppendorf AG, Hamburg, Germany). Then, the PCR was run according to the following protocol: denaturation at 94 °C for 5 min, followed by 35 cycles shared in denaturation at 94 °C for 1 min, annealing at 62 °C for 1 min, extension at 72 °C for 1 min, and final extension at 72 °C for 10 min. The cycler used was Mastercycler^®^ Gradient (Eppendorf AG, Hamburg, Germany).

After the PCR, the products were separated by electrophoresis on 2% Agarose gel (GellyPhor, Euroclone, UK). A 100 bp DNA marker was also run with the PCR products (Invitrogen, Carlsbad, CA, USA). Finally, the separated products were digested with 5 U of *MnlI* enzyme (New England Biolabs, Beverly, MA, USA) and 2 U of *RsaI* enzyme (New England Biolabs, Beverly, MA, USA). Specifically, the digestion was conducted at 37 °C for 2 h on a 30 μL volume composed as follows: 20 μL of PCR products, 0.3 μL of BSA 100 μL/mL, 3 μL of 1× buffer (which was a solution composed of 10 mM of Tris-HCl, 50 nm of NaCl, 10 mM of MgCl_2_, 1 mM of dithiothreitol, and pH 7.9 for *MnlI*; 10 mM of Bis-Tris-propane-HCl, 10 mM of MgCl_2_, 1 mM of dithiothreitol, and pH 7.0 for *RsaI*). Subsequently, a deactivation process at 65 °C for 20 min was carried out. The fragments thus obtained were separated by electrophoresis on 4% Agarose gel (GellyPhor, Euroclone, UK). A 50 bp DNA marker was also run with the digested fragments (Invitrogen, Carlsbad, CA, USA). Genotyping was conducted on all the samples.

### 2.3. Statistical Analysis

Allele and genotype frequencies were determined through a direct count of the observed genotypes. The chi-squared test was used to determine the Hardy–Weinberg equilibrium of the mutation (Genepop version 4.2).

## 3. Results

In all breeds involved in the study, the amplification of genomic DNA by PCR and the use of primers designed by Messer et al. resulted in the production of 824 bp fragments [23].

**Figure 1:**
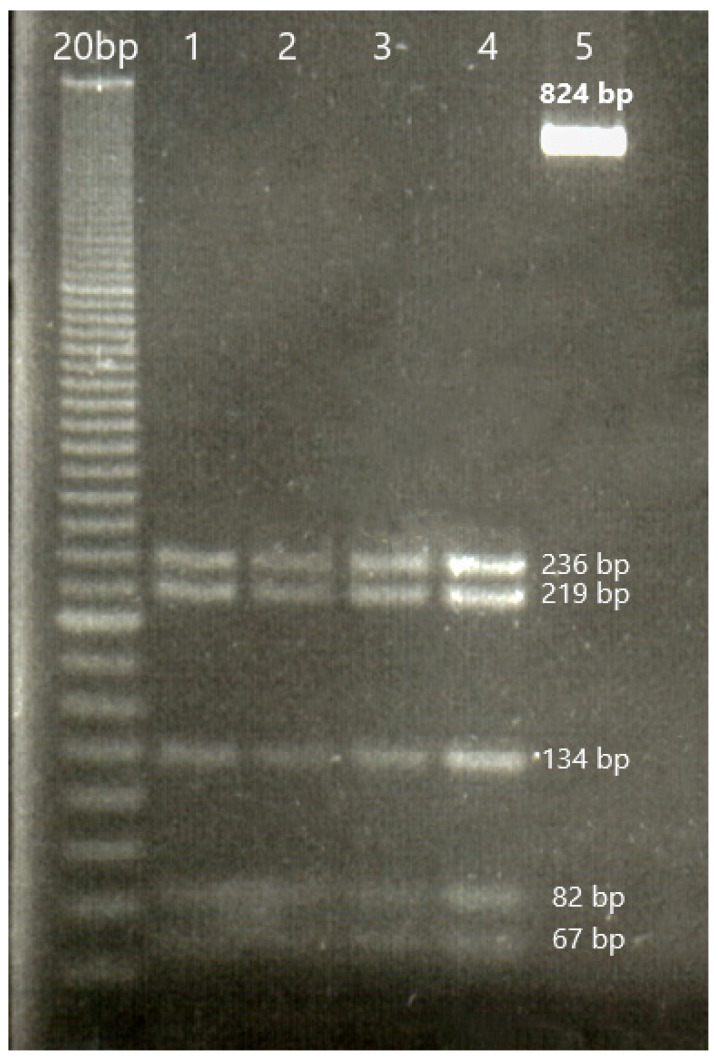
Electrophoresis of digestion with *MnlI* of the II exon of *MTNR1A* gene in the investigated goat breeds (4.0% agarose gel). DNA ladder 20 bp; lines one to four are M/M genotype, and line five is the entire fragment amplified by PCR. The fragments with a length of 22, 28, and 36 bp are too small to be evidenced.

Following digestion with the *MnlI* enzyme, the formation of eight fragments with a length of 219, 36, 67, 236, 22, 28, 82, and 134 bp, based on the order of the cleavage sites along the sequence of the amplified fragments, was evidenced for all the goats investigated. The cleavage sites were located at 219, 255, 322, 558, 580, 608, and 690 positions, respectively. No polymorphisms were found in the g.17355452 (G > A) position, and consequently, the fragment with a length of 303 bp was divided into two parts of 67 and 236 bp. Indeed, the presence of a guanine nucleobase (G) in the g.17355452 position was responsible for the cleavage of the *MnlI* enzyme (i.e., M allele) and the fragmentation of the 303 bp fragment, while an adenosine nucleobase (A) would have caused the absence of fragmentation (m allele). All the goats enrolled in our study had the M/M genotype (Table 2 and Figure 1).

**Figure 2:**
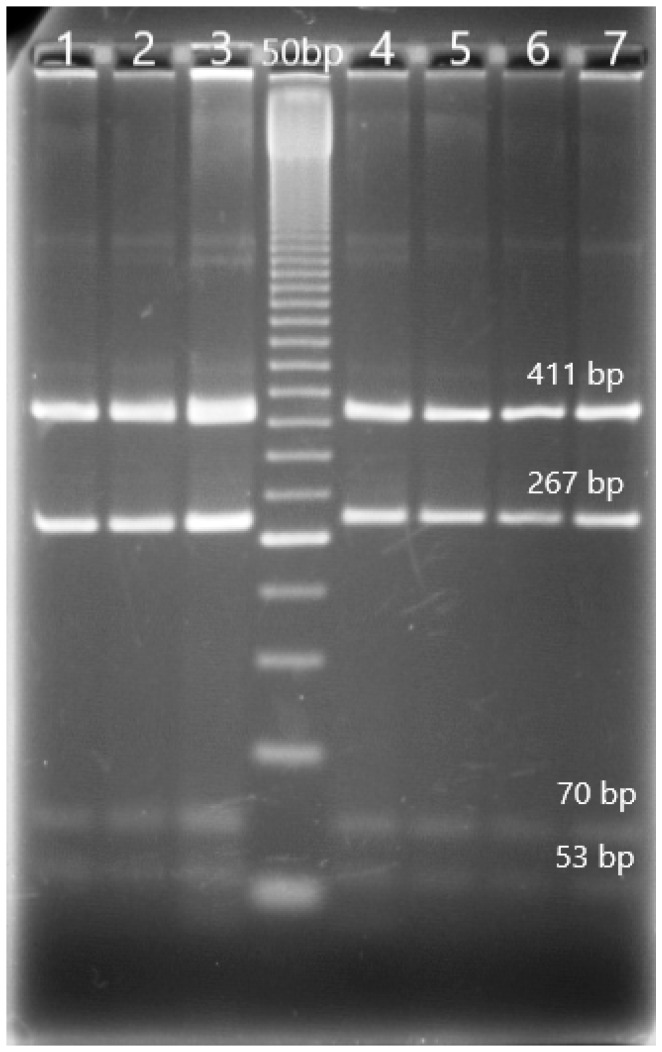
Electrophoresis of digestion with *RsaI* of the II exon of *MTNR1A* gene in the investigated goat breeds (4.0% agarose gel). DNA ladder 50 bp; lines one to three and four to seven are R/R genotype. The fragment with a length of 23 bp is too small to be evidenced.

Digestion of the 824 bp-amplified fragment with the restriction enzyme *RsaI* resulted in the production of five fragments of 53, 267, 23, 411, and 70 bp length, based on the order of the cleavage sites along the sequence of the amplified fragments, for all the investigated goats. The cleavage sites identified with the *RsaI* enzyme were four, at positions 53, 320, 344, and 755, respectively. Again, no polymorphisms were found in the g 17355458 (C > T) position, and consequently, the fragment with a length of 290 bp was divided into two parts of 267 and 23 bp. Indeed, the presence of a cytosine nucleobase (C) in the g 17355458 position was responsible for the cleavage of the *RsaI* enzyme (i.e., R allele) and the fragmentation of the 290 bp fragment, while a tyrosine nucleobase (T) would have caused the absence of fragmentation (r allele). All the goats enrolled in our study had the R/R genotype (Table 3 and Figure 2).

## 4. Discussion

The fragment of 824 bp found in all goat breeds following the amplification of exon II′s genomic DNA is consistent with what has already been reported since the first investigation on the sheep genome [23], followed by the few investigations on the goat species [38,46,47,50] using the primers by Messer et al. [23].

The *MTNR1A* gene showed the same cleavage sites for the *MnlI* and *RsaI* enzymes in Frisa Valtellinese, Orobica, Lariana, and Camosciata delle Alpi goats. Specifically, our results showed the existence of seven cleavage sites for *MnlI* and four for *RsaI*. This is in agreement with what has been previously reported in studies both on sheep and goats [37,38,46,50]. Our result is only partially consistent with the study by Chu et al., which identified just six cleavage sites for *MnlI* in five Chinese native breeds and a cosmopolitan goat breed [47]. Regardless, in our study, none of the cleavage sites were polymorphic.

Regarding the fragments produced by *MnlI* digestion, our findings are consistent with what was reported by Migaud et al. [46], who conducted the same experimental plan on Camosciata delle Alpi and Creole goats. Interestingly, also in that case, no polymorphic cleavage sites were reported in either breed, despite the former being a seasonal estrous breed and the latter a year-round estrous breed, and differences between the two breeds were expected, based on results in sheep species. In goats, the same unexpected result was also obtained by Chu et al. and Carcangiu et al. [38,47]. In sheep species, a polymorphism at the g.17355452 position was found following *MnlI* enzymatic digestion, which showed a strong link with reduced reproductive seasonality in different Mediterranean breeds. This was shown to be associated with a mutation at the g.17355358 position, which led to an amino acid substitution of Val to Ile, causing a modification in the melatonin signal transmission [30].

Concerning the polymorphism identified by the restriction enzyme *RsaI*, in our study, none of the four cleavage sites were polymorphic. While the identified sites are in concordance with what has been reported in the literature for the goat species [38,47,50], the absence of any polymorphism was an unexpected result. In fact, Chu et al. reported the presence of a polymorphic cleavage site following digestion with the above-mentioned enzyme [47]. The allele associated with the presence of the cleavage site was termed R, while the absence of it was represented by the letter r. In the study by Chu et al., six goat breeds were considered, two of which were year-round estrous. The latter breeds showed an association with the R/R genotype, while native Chinese breeds with marked reproductive seasonality were associated with an R/r genotype. No goats showed the r/r genotype, from which a low frequency of the r allele in the goat species is assumed [47]. The same results were also found in two different Turkish local goat breeds, but neither of them showed a r/r genotype [50]. Subsequently, an investigation by Carcangiu et al. confirmed the presence of the polymorphic site identified at position 53 by the restriction enzyme *RsaI* [38] in the goat species. In this study, four cosmopolitan goat breeds (Saanen, Camosciata delle Alpi, Maltese, and Nubiana), primarily reared for milk production, were examined in addition to the Sarda breed. The Sarda goat is a local Italian breed raised for the production of milk, which is then processed for traditional dairy production. In contrast to cosmopolitan breeds, the Sarda has undergone less breeding selection by humans. The results showed the presence of the r allele exclusively in the Sarda breed, in association with a more marked reproductive seasonality [38]. For these reasons, considering the marked reproductive seasonality of the breeds examined in our study, coupled with their origin in regions at higher latitudes than the Sarda breed, and the limited artificial selection for reproductive activity (at least in the local Italian breeds enrolled), the absence of polymorphism in relation to digestion with the restriction enzyme *RsaI* represents a most unexpected result. Camosciata delle Alpi is an exception, with our results being consistent with the study by Carcangiu et al. [38]. One hypothesis, mentioned by Carcangiu et al. [38], is that the absence of polymorphism may be linked to the macro group of goat breeds that evolved during human migrations. Specifically, breeds of the Alpine–European strain would appear to show no polymorphism, as confirmed by our study, whereas breeds belonging to the Mediterranean–African [38] or Asian–Middle Eastern [47,50] strains do.

Finally, it is interesting to compare our results on the goat species with the most recent advances in the ovine species. Indeed, even in sheep, the most investigated SNPs are rs430181568 (at position g.17355452) and rs406779174 (at position g.17355458) [30]. These are the same polymorphisms that were specifically investigated in our study. In sheep, the genotypes associated with these polymorphisms occur in variable percentages in sheep populations depending on the breed considered, and their effects on reproductive performance also differ according to the breed. What is consistent, however, in all the studies conducted to date, is that the SNP at position g.17355452 is linked with the SNP rs407388227 G > A at position g.17355358, to such an extent that they can be considered a single marker [30]. This latter SNP causes amino acid changes, with the substitution of Val with Ile in the gene sequence, which is responsible for the MT1 receptor signaling modification [54]. This fact has been suggested as being responsible for the influence of the polymorphism in g.17355452 on reproductive traits in sheep. However, this SNP, in the goat breeds investigated to date, has always been found to be non-polymorphic, therefore determining the need to further investigate the sequence of the *MTNR1A* gene in order to determine more precisely the relationship existing between the gene and the reproductive traits.

## 5. Conclusions

In conclusion, our results showed for the first time the absence of polymorphism of the melatonin receptor gene *MTNR1A* identified by the restriction enzymes *MnlI* and *RsaI* in the three Italian goat breeds (i.e., Frisa Valtellinese, Orobica, and Lariana) and in the transboundary goat breed Camosciata delle Alpi. This represents an unexpected result for the above-mentioned reasons. We hypothesize that this result could be due to the different spatiotemporal evolution of goat breeds around the world, which has led to genetic differences among macro groups of breeds. Future research is essential to better investigate these aspects and to validate our findings. Additionally, upcoming studies should encompass a larger number of animals. However, the following must be considered: the decline in local breed populations due to their progressive abandonment by farmers, and the close parental relationship between individuals of the same breed given the exclusive use of natural mating through the exchange of bucks between breeders. Moreover, further investigations on other autochthonous goat breeds of the peninsula, which are traditionally raised at different latitudes, could provide valuable insight into the role of the *MTNR1A* gene polymorphism in regulating reproductive seasonality. Further analyses, including genetic investigations, could help broaden knowledge in this area. Finally, parallel analyses conducted on the ancestor of the domestic goat, i.e., *Capra aegagrus*, could be very interesting, following the example of what has already been conducted on the sheep species *Ovis Gmelini Musimon*, a feral subspecies of primitive domestic sheep [55].

## Figures and Tables

**Table 1:** Litter size per breed.

Litter Size	Breed			
	Frisa Valtellinese	Orobica	Lariana	Camosciata delle Alpi
1	18 (62.1%)	23 (100.0%)	24 (82.8%)	5 (18.5%)
2	11 (37.9%)	0 (0.0%)	5 (17.2%)	20 (74.1%)
3	0 (0.0%)	0 (0.0%)	0 (0.0%)	2 (7.4%)
Total	29	23	29	27

**Table 2:** Allelic and genotypic frequencies in the analyzed goat breeds of M and m alleles.

Breed	M	m	M/M ^1^ (%)	M/m ^2^ (%)	m/m ^3^ (%)
Frisa Valtellinese	1.00	0.00	100	0	0
Orobica	1.00	0.00	100	0	0
Lariana	1.00	0.00	100	0	0
Camosciata delle Alpi	1.00	0.00	100	0	0

^1^ M/M = G/G; ^2^ M/m = G/A; ^3^ m/m = A/A.

**Table 3:** Allelic and genotypic frequencies in the analyzed goat breeds of R and r alleles.

Breed	R	r	R/R ^1^ (%)	R/r ^2^ (%)	r/r ^3^ (%)
Frisa Valtellinese	1.00	0.00	100	0	0
Orobica	1.00	0.00	100	0	0
Lariana	1.00	0.00	100	0	0
Camosciata delle Alpi	1.00	0.00	100	0	0

^1^ R/R = C/C; ^2^ R/r = C/T; ^3^ r/r = T/T.

## Data Availability

Data will be made available upon reasonable request.
